# Selective biofunctionalization of 3D cell-imprinted PDMS with collagen immobilization for targeted cell attachment

**DOI:** 10.1038/s41598-022-17252-6

**Published:** 2022-07-27

**Authors:** Mahrokh Babaei, Shahin Bonakdar, Bahram Nasernejad

**Affiliations:** 1 Department of Chemical Engineering, Amirkabir University of Technology (Tehran Polytechnic), Tehran, Iran; 2 National Cell Bank Department, Pasteur Institute of Iran, Tehran, Iran

**Keywords:** Biomaterials, Biomimetics, Nanobiotechnology, Regenerative medicine, Stem-cell biotechnology, Tissue engineering, Biomedical engineering, Chemical engineering, Nanobiotechnology, Nanomedicine, Nanoscale materials, Other nanotechnology, Mesenchymal stem cells, Stem-cell niche, Biotechnology, Engineering, Nanoscience and technology, Materials science, Biomaterials, Biomaterials - cells

## Abstract

Cell-imprinted polydimethylsiloxane substrates, in terms of their ability to mimic the physiological niche, low microfabrication cost, and excellent biocompatibility were widely used in tissue engineering. Cells inside the mature cells' cell-imprinted PDMS pattern have been shown in previous research to be capable of being differentiated into a specific mature cell line. On the other hand, the hydrophobicity of PDMS substrate leads to weak cell adhesion. Moreover, there was no guarantee that the cells would be exactly located in the cavities of the cells' pattern. In many studies, PDMS surface was modified by plasma treatment, chemical modification, and ECM coating. Hence, to increase the efficiency of cell-imprinting method, the concavity region created by the cell-imprinted pattern is conjugated with collagen. A simple and economical method of epoxy silane resin was applied for the selective protein immobilization on the desired regions of the PDMS substrate. This method could be paved to enhance the cell trapping into the cell-imprinted pattern, and it could be helpful for stem cell differentiation studies. The applied method for selective protein attachment, and as a consequence, selective cell integration was assessed on the aligned cell-imprinted PDMS. A microfluidic chip created the aligned cell pattern. After Ar^+^ plasma and APTES treatment of the PDMS substrate, collagen immobilization was performed. The immobilized collagen was removed by epoxy silane resin stamp from the ridge area where the substrate lacked cell pattern and leaving the collagen only within the patterned areas. Coomassie brilliant blue staining was evaluated for selective collagen immobilization, and the collagen-binding stability was assessed by BCA analysis. MTT assay for the evaluation of cell viability on the modified surface was further analyzed. Subsequently, the crystal violet staining has confirmed the selective cell integration to the collagen-immobilized site on the PDMS substrate. The results proved the successfully selective collagen immobilization on the cell-imprinted PDMS and showed that this method increased the affinity of cells to attach inside the cell pattern cavity.

## Introduction

Providing a culture medium similar to the natural niche of cells causes the appropriate conditions in comparison with a traditional cell culture plate for cell culturing. Cellular activities may be regulated and the circumstances for adhesion, growth, migration, and differentiation can be provided by simulating the physicochemical aspects of the cellular microenvironment, such as extracellular matrix (ECM) proteins and topographical features^1–4^. Polymer surfaces were widely used to transfer molecular and cellular patterns^5,6^. Polydimethylsiloxane (PDMS) is one of the main polymers for cell imprinting-based studies and cell behavior investigations^3,7,8^. Using cells to cell-imprint, create micro-/nano structures with active areas which can get engaged in the cell–surface interactions. Co-existence of patterned and flat surfaces on the cell-imprinted substrate in terms of non-uniform cell spreading makes a problem for previous studies on the cell-imprint technique. The seeded cells will be attached both inside the cell-imprinted cavities and flat areas. Because of this diversity, cell signaling is altered and cell fates are diverged. Consequently, this issue may be resolved by placing cells in the cell-imprinted cavities in a selective manner. We developed a novel method to eliminate the stray cells beyond the defined patterns. First, the whole of the cell-imprinted surface was biofunctionalized by collagen. Then, using an epoxy silane resin stamp, the immobilized collagen was removed from the undesired area (smooth area) while the cell-imprinted pits remained covered with collagen. In this field, one of the most commonly used epoxy silane resins for protein immobilization is (3-Glycidyloxypropyl) trimethoxysilane (GPTMS) which is applied in this work^9^. Immobilization of protein and enzyme on the epoxy-activated substrate was performed in many studies^10–13^. Protein immobilization by covalent attachment may be achieved on these supports because they provide the necessary matrices. Nucleophilic groups on the protein surface (such lysine, cysteine, histidine, and tyrosine) may rapidly react with epoxy groups, but carboxylic groups take longer. Also, these groups may react with carboxylic groups to form an ester bond, although at a slower fashion^14^. However, incubation of the epoxy-activated supports with proteins under alkaline conditions achieves the highest intensity of multipoint covalent linkages^15,16^, and the neutral pH condition causes the formation of a few covalent bonding between the protein and the epoxy groups^14,17^. Thus, protein immobilization is performed from pH 4 to 10 by combining carboxyl-epoxy or amino-epoxy. The integration of epoxy silane resin with collagen was applied to remove the collagen from undesired regions of cell-imprinted PDMS. Selective collagen integration and preserving collagen functionality on the PDMS surface are our main targets. Hence, regarding collagen manufacture’s protocol, collagen at neutral pH condition was coated on the cell-imprinted PDMS, afterward, the epoxy silane resin stamp method was applied at the same PH condition. The efficiency of this method was investigated on the aligned cell-imprinted surface created by a microfluidic chip and the ADSCs were cultured on the designed substrate. The results showed a successful cell attachment inside the desired sites on the PDMS substrate. Therefore, in addition to cell differentiation studies, this method can be used in microfluidic, tissue engineering, and modified biomaterial surface studies.

## Materials and methods

### Cell culture

The ethics committee approved all the experiments of the Pasteur Institute of Iran. Human umbilical vein endothelial cells (HUVECs, Cell Bank of Pasteur institute of Iran) were used to prepare the aligned cell pattern. After obtaining informed consent, the adipose-derived stem cells (ADSCs) were isolated from adipose tissue withdrawn from healthy 20 years old human bodies and were used for culture on modified PDMS. First, adipose tissue was washed three times in phosphate-buffered solution (PBS) with 3% Penicillin/Streptomycin (Sigma, USA), then they were cut into 1–2 mm pieces and digested in 0.02 mg/ml collagenase type I (Sigma, United States) at 37 °C for 2 h. The solution was passed through a 75 μm filter to remove undigested tissue, followed by neutralization of the enzyme with Gibco Dulbecco's Modified Eagle Medium (DMEM, Gibco) containing 10% fetal bovine serum (FBS, Gibco). Finally, they were centrifuged at 1300 rpm for 5 min to separate the cellular pellets. The obtained solution consisting of ADSCs was transferred to the culture medium consisting of DMEM/Ham’s F12 supplemented with 100 μg/ml streptomycin, 100 U/ml penicillin, and 10% Fetal bovine serum (FBS) and was incubated at 37 °C in a 5% CO_2_ incubator^18^. After 24 h, the non-adherent cells and debris were discarded, and a new culture medium was added. ADSCs in the third passage were used for cell seeding.

### Preparing the aligned cell-imprinted PDMS substrate using a microfluidic chip 

**Figure 1:**
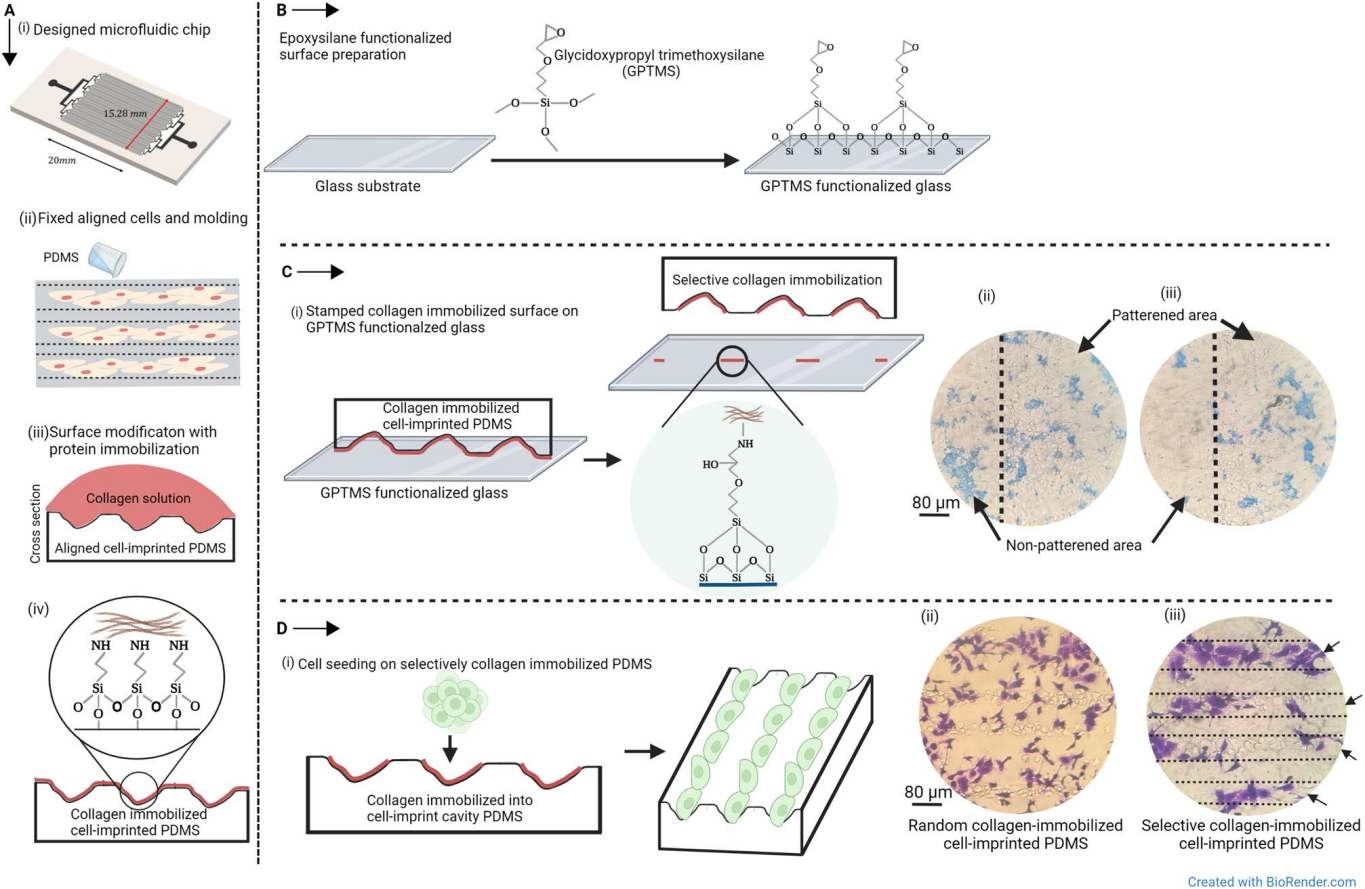
Schematic illustration of (**A**-i, ii) creating aligned cell-imprinted PDMS substrate by micro-channels, (**A**-iii, iv) modification of aligned cell imprinted PDMS surface by collagen immobilization, (**B**) functionalization of the glass substrate by GPTMS as stamp layer, (**C**-i) preparing the selective collagen-immobilization into the desired regions of PDMS substrate by using epoxy silane resin stamp method, (**C**-ii) Coomassie brilliant blue staining of the aligned cell-imprinted PDMS surface with random and (**C**-iii) selective collagen immobilization, (**D**-i) cell seeding on selective collagen-immobilized PDMS, (**D**-ii) crystal violet staining of cultured ADSCs on the random and (**D**-iii) selective collagen-immobilized cell-imprinted PDMS after 24 h.

The aligned cell-imprinted substrate was prepared better to display the protein immobilization into the desired regions. The aligned cell-imprinted substrate by microfluidic chip was created based on the previous study^19^. In brief, a set of 128 micro-channels with a length of 20 mm and a depth of 50 μm was considered. The micro-channels have a 40 μm width and the ability to accommodate about 3 × 10^6^ HUVEC cells in aligned lines. Microfluidic chip after washing with ethanol and sterilization in the autoclave was placed on a cell culture plate with channels side facing down. HUVEC cell solution with the concentration of 6 × 10^6^ in 150 μl of culture medium DMEM/Ham’s F12 was injected into the chip at a flow rate of 50 μl/min by a syringe pump. After filling up the microchannel with the cells, the cell culture plate and microfluidic chip were incubated for 7 h for complete cell adhesion to the cell culture plate. At this point, we took out the microfluidic chip and washed the cells in PBS before fixing them in a 4% glutaraldehyde solution for 1 h. To transfer the ordered arrangement of the fixed cells to the PDMS, a layer of PDMS 10:1 was poured over them. After peeling the cured PDMS away from the fixed cells, a 1 M NaOH solution wash was used to remove any leftover cells or residues (Fig. 1A-i,ii).

### Selective biofunctionalization of PDMS substrate by collagen immobilization

PDMS substrate consists of aligned cell pattern was treated with argon plasma (Harrick Plasma-PDC 32G) for 3 min at the pressure of 0.3 mbar followed by their immersion in 3-Aminopropyl triethoxysilane (APTES) (Sigma-Aldrich, USA) 10% in ethanol at 50 °C for 2 h. After removing APTES solution and washing twice with nuclease-free water, the samples were incubated with 20 μg/ml of bovine collagen (Nanozistaraye, Pasteur Institute, Iran) solution on a rocker shaker for 2 h and then were stored at 4 °C overnight followed by removing the collagen solution and washing with nuclease-free water. The collagen-coated PDMS substrate was stamped on an epoxy silane-modified slide to remove the collagen from the undesired regions from the undesired regions. Eventually, the resulting substrate was sterilized at ethanol 70% and was placed under UV light for 45 min. The non-selective collagen-immobilized substrate whose entire surface was coated with collagen was used as the negative control. Schematic presentation of the surface modification of PDMS is depicted in Fig. 1A-iii,iv, and the method for selective detachment of immobilized collagen showed in Fig. 1C-i.

### Epoxy silane activated surface preparation 

Untreated slides were washed with ethanol and then etched by immersion in 10% NaOH at 25 °C for 1 h, followed by sonication for 15 min in 10% NaOH. Later, it was rinsed four times in water, washed twice in ethanol, and derivatized in the coating solution: 2.5% (3-glycidoxypropyl) trimethoxysilane (GPTMS) and 10 mM acetic acid in ethanol at 25 °C for 1 h, again followed by a sonification step. Subsequently, they were washed thoroughly with ethanol and dried^20^. Finally, they baked in a vacuum oven at 50 °C for 1 h and were ready to use as stamp surfaces (Fig. 1B).

### Evaluation of the selective protein adhesion

We performed Coomassie brilliant blue staining assay to identify the selective collagen adhesion into the pits on the PDMS substrate. The random and selective collagen-immobilized PDMS, after washing three times with PBS were treated with Coomassie brilliant blue solution (1% w/v Coomassie brilliant blue in 50% methanol and 10% glacial acetic acid) for 1 h with shaking at 25 °C. After the mentioned time, the Coomassie brilliant blue solution aspirated off, the samples were washed three times with deionized water^21^. Afterwards, the samples were imaged under an optical microscope (BEL, INV2, Italy). Images were analyzed with the ImageJ program, and the area of the stained region was measured. Afterward, the position of immobilized collagen and the percentage of the covered area was quantified.

### Stability of immobilized collagen on the PDMS substrate

To check the stability of immobilized collagen on the PDMS substrate, a micro-BCA protein assay kit (Thermo Scientific, USA) was used. Collagen retention on the random and selective collagen-immobilized PDMS was measured on days 0, 7, and 14 after immobilization and applying the epoxy silane stamp method. For this purpose, the samples (4 cm^2^) were stored in sterilized 1× PBS buffer, pH 7.2 at 37 °C and 5% CO_2_. In order to get rid of any free-floating proteins, samples were treated with 0.5% Tween 20 (Sigma-Aldrich, USA) for 30 min and then washed twice with nuclease-free water at the indicated times. Collagen retention on the surfaces was evaluated using the kit's specified procedure. The absorbance of samples was measured at 562 nm with the Multiskan Spectrum microplate reader (Thermo Scientific, Singapore)^22^. Hence, the final amount of remaining collagen on the substrates was calculated based on the initial concentration of collagen, which was 20 µg/ml.

### Evaluation of the cell viability on the random and selective collagen-immobilized PDMS substrate

To evaluate the effect of the epoxy silane stamp method and the process for preparing the collagen-immobilized substrate on cell viability, the MTT (3-[4,5-dimethythiazol-2-yl]-2,5-diphenyltetrazolium bromide; Sigma) assay was performed. Random and selective collagen-immobilized polydimethylsiloxane (PDMS) substrates were submerged in 70% ethanol for 30 min, dried under a laminar hood, and then irradiated with UV light for 45 min before being seeded with cells. ADSCs were sown on the sterile samples at a density of 104 cells/well. A cell-imprinted PDMS without collagen immobilization was used as a control. The samples were incubated for 1, 3, and 7 days at 37 °C with 5% CO2. At mentioned time points, the MTT solution at a concentration of 0.5 mg/ml was added to each well, then cells were stored in the incubator for 4 h at 37 °C. After the formation of formazan crystal, the medium was removed, and the crystals were dissolved in isopropanol. The plate was placed in the orbital shaker for 15 min to enhance the dissolution process. Optical density was measured by the ELISA reader (ELX800 Universal Microplate Reader, BIO-TEK Instruments, USA) at 570 nm^23^.

### Evaluation of ADSCs' desire to attach to the collagen-immobilized PDMS substrate

Crystal violet staining was performed to indicate the affinity of cell attachment to the selective collagen-immobilized region. The random and selective collagen-immobilized PDMS substrates were immersed in 70% ethanol for 30 min, and dried under a laminar hood followed by UV light irradiation for 40 min before ADSCs seeding. We seeded 1 × 10^4^ ADSCs per cm^2^ in culture medium DMEM/Ham's F12 (3:1 ratio) and 10% (v/v) FBS on the samples and they were incubated at 37 °C with 5% CO_2_ overnight. A schematic presentation of cell seeding on the selective collagen-immobilized PDMS substrate was shown in Fig. 1D-i. After 24 h of culture, the cells were fixed with 4% glutaraldehyde solution for 24 h. Thus, fixed cells were stained with 1% crystal violet in 50% methanol for 10 min at 25 °C, then washed with distilled water. Stained viable cells cultured on the substrates were observed under an optical microscope (BEL, INV2, Italy)^24,25^. Images were analyzed with the ImageJ program. The location of attached cells and the total area covered by cells were quantified and statistically analyzed.

### Statistical analyses

Statistical analysis was performed using OriginLab software. Data statistically were analyzed using one-way analysis of ANOVA, followed by Tukey multiple comparison tests to determine the statistical significance. A *p* < 0.05 was considered statistically significant.

### Ethical standard

In vitro and in vivo experiments were registered at Pasteur Institute of Iran and confirmed by its ethics committee. This project was supported by grant No. 1582 in Pasteur Institute of Iran.

## Results and discussion

### Coomassie brilliant blue staining verified the selective immobilization of collagen on PDMS

**Figure 2:**
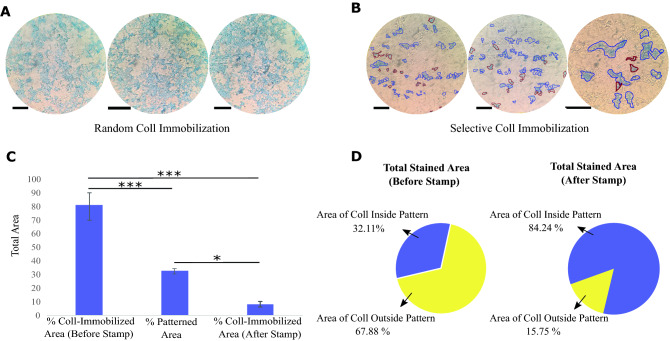
Coomassie brilliant blue staining of the aligned cell-imprinted PDMS surfaces (**A**) before and (**B**) after using the stamp method. Immobilized collagen inside the pattern with a blue border and outside pattern regions with a red border was marked (Scale bar = 80 μm). (**C**) The total area of the immobilized collagen before and after the stamp method compares with the total patterned area. (**D**) The ratio of immobilized collagen inside to outside of patterned regions before and after the stamp method. The area is measured by ImageJ software, and the graph represents the mean ± SD. *p value of < 0.05 and ***p value of < 0.001 between two groups (N = 6).

The result of Coomassie brilliant blue staining demonstrated that, after using the epoxy silane resin stamp method significantly, collagen remains only inside the concavity regions created by cell-imprinting. Coomassie brilliant blue staining was not seen in areas of the ridges where collagen had been removed using an epoxy silane resin stamp (Fig. 1C-ii,iii). Coomassie brilliant blue staining was used to demonstrate the before and after effects of the selection technique (Fig. 2A,B). Figure 2C shows the percentage of total collagen immobilized area before and after using the selective method. Comparison between the collagen immobilized area and total patterned area, confirmed the significantly less difference (*p* value of < 0.05) after the stamp method. Moreover, Fig. 2D shows the ratio of immobilized collagen inside patterned to outside patterned regions. This amount was 32.11 to 67.88% for samples before the stamp procedure. While after using the stamp method, this ratio has a notable growth and reached 84.24 to 15.75%. The results show that although the patterned regions are 32.67% of the total area, the ratio of collagen inside patterned to outside patterned has increased from 0.47 to 5.34 after using the epoxy silane stamp method. Therefore, the Coomassie brilliant blue staining assay revealed the successful selective collagen immobilization into the cell-imprinted pits.

As previously proven, the epoxy silane resin can immobilize the proteins on support surfaces^26–28^. Hence, the collagen-immobilized PDMS was stamped on the GPTMS functionalized slide and caused removing collagen from the ridge regions via collagen attachment to GPTMS-functionalized glass. Removing the collagen from the PDMS substrate and transferring it to the epoxy silane-activated supports showing a stronger covalent bond between collagen and epoxy silane functionalized glass compared to silanized PDMS.

**Figure 3:**
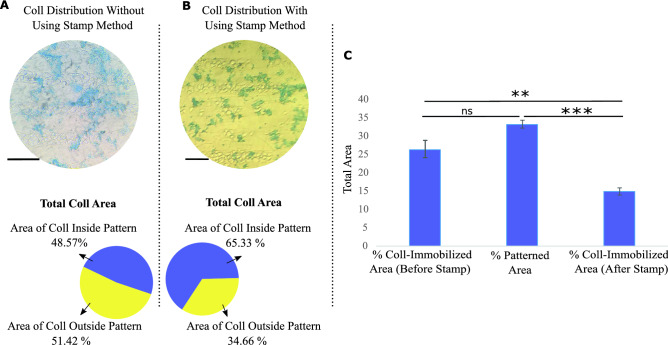
Illustration of the collagen distribution and the ratio of collagen inside to the outside pattern after 14 days by Coomassie brilliant blue staining on the samples (**A**) without and (**B**) using the stamp method (Scale bar = 80 μm). (**C**) The total area of the collagen immobilized before and after the stamp method compared to the total patterned area after 14 days. The graph represents the mean ± SD. **p value of < 0.01, ***p value of < 0.001, and (ns) indicates a statistically non-significant result between two groups (N = 3).

Another important factor is the ability of immobilized collagen to preserve the arrangement over time. After 14 days, the samples without and with the epoxy silane stamp method were stained by Coomassie brilliant blue for evaluation of the collagen distribution. Figure 3A shows the random distribution of collagen on the PDMS surface for the samples without using the stamp method. The obtained results from the samples after using the stamp method confirmed that the immobilized collagen can preserve its aligned arrangement, and no noticeable displacement was observed (Fig. 3B). Figure 3C displayed the percentage of the collagen-immobilized area before and after the epoxy stamp compared to the total patterned area. Considering that the patterned regions are 33.37% of the total surface and as known from Fig. 2D for random sample the immobilized collagen inside and outside patterned regions was 32.11% and 67.88%, respectively, while these amounts were measured to be 48.67% and 51.42% after 14 days, its obvious most of the removed collagen over time was from non-patterned regions.

### BCA analysis confirmed the stability of collagen-binding after applying the epoxy silane stamp method

**Figure 4:**
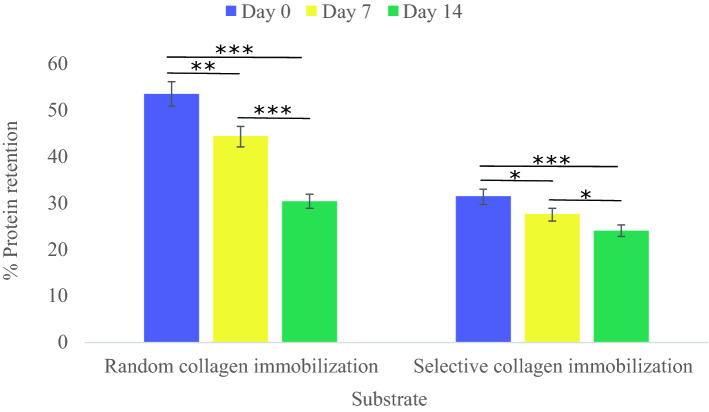
Micro BCA assay for calculating the amount of attached and retained collage on the random and selective collagen-immobilized PDMS substrates on 0, 7, and 14 days. The graph represents the mean ± SD. *p value of < 0.05, **p value of < 0.01, and ***p value of < 0.001 between two groups.

Collagen attachment and retention on aligned cell-imprinted PDMS surface before and after the epoxy silane resin stamp method were analyzed by micro BCA as shown in Fig. 4. The figure shows the percentage ratio of attached and retained collagen compared to the initial concentration (20 µg/ml) on days 0, 7, and 14. The data obtained from the sample before applying the stamp method are as follows 53.4%, 44.23%, and 30.26% on days 0, 7, and 14 m respectively. While according to the collected data, the remaining collagen decreases after applying the stamp method and was measured to be 31.3%, 27.5%, and 23.9% on days 0, 7, and 14. The difference between 53.4 and 31.3% for the samples on day 0 indicates the removed collagen from the ridge area by epoxy silane resin. After 14 days, random collagen coatings had lost 43.33% of their immobilized collagen compared to the 23.64% loss seen with selective collagen immobilization. However, with time the amount of immobilized collagen declined in both samples, which could be in terms of the protein degradation under incubation conditions (37 °C and 5% CO_2_)^29^. The selective collagen immobilized sample presented more stability of immobilized collagen compared to day 0. Moreover, based on the Coomassie brilliant bluestaining results of samples after 14 days, the ability of the selective collagen-immobilized sample to preserve the aligned arrangement is in line with these results. Therefore, it is concluded that the covalent bonding between the collagen molecules and the patterned area is more stable than the ridge area.

### MTT assay confirmed the biocompatibility of the selective collagen-immobilized method

**Figure 5:**
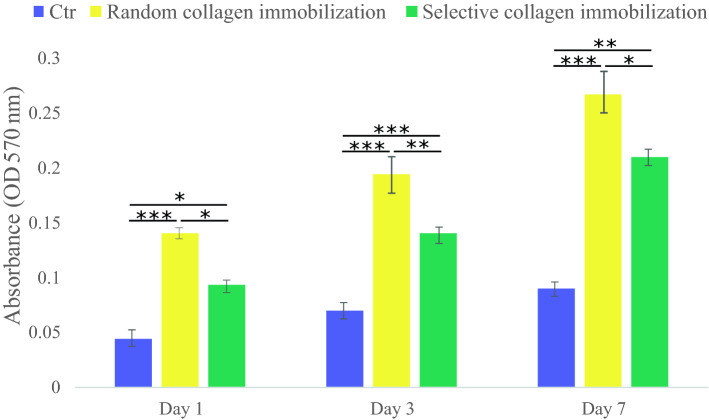
Cell viability of ADSCs on the random and selective collagen-immobilized PDMS substrates. Cell-imprinted PDMS without collagen immobilization was used as a control. The data shows the mean ± SD. *p value of < 0.05, **p value of < 0.01, and ***p value of < 0.001 between two groups.

To evaluate the applied protocol on cell proliferation and cell viability: the MTT assay measures for 1, 3, and 7 days after cell culture, and Fig. 5 shows the result. Both random and selective collagen-immobilized PDMS substrates showed significantly more cell viability compared to plain PDMS as a control. The surface hydrophobicity of PDMS renders it unsuitable for use in cell culture. The selective collagen-immobilized PDMS substrate had a lower number of viable cells than the random collagen-immobilized PDMS substrate at all time periods. This decrease occurred because the accessible area consisting of collagen immobilization after the epoxy stamp was reduced. Therefore, it is no surprise to observe less cell viability and proliferation on the selective collagen-immobilized PDMS, and it was in terms of removing collagen from some parts of the PDMS surface. Additionally, the result shows the growth cycle of ADSCs on the selective collagen immobilized surface has slowed, and the differentiation paths started that require specific analysis to confirm this argument.

### Crystal violet staining showed more affinity of ADSCs to attach inside the cell patterned area after applying the stamp method

**Figure 6:**
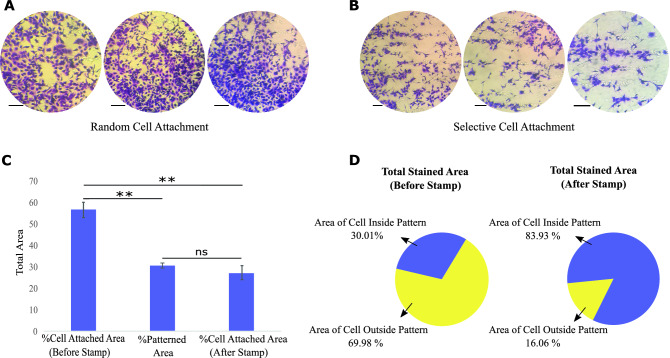
Crystal violet staining of ADSCs seeded on the collagen-immobilized PDMS substrate (**A**) before and (**B**) after using the stamp method (Scale bar = 80 μm). (**C**) The comparison between the total area of the attached cell before and after the stamp method with the total patterned area. (**D**) The ratio between the attached cells inside and outside the patterned regions before and after the stamp method. The measured area was measured by ImageJ software. The graph represents the mean ± SD. **p value of < 0.01 and (ns) indicates a statistically non-significant result between the two groups (N = 6).

Figure 1D-ii,iii show that crystal violet staining was carried out to observe the spread arrangement of ADSCs on different PDMS surfaces. As expected, crystal violet staining of ADSCs implanted on the random collagen-immobilized PDMS substrate revealed random cell distribution. Figure 6A,B illustrate when cell adhesion orientation synchronizes with the aligned cell pattern on the selectively immobilized collagen-containing substrate. Figure 6C illustrates the proportion of total cell area before and after the selective approach was used. By comparing these values with the total patterned area, it was found that after applying the stamp method, there is no significant difference between the percentage of area for attached cells and total patterned regions. Therefore, it can be concluded that the stamp method guided the selective cell attachment. Moreover, Fig. 6D shows the ratio between attached cells inside and outside patterned regions. These amounts were 30.01 to 69.98% for samples before the stamp method, and after using the stamp method, they significantly increased and reached around 83.93 to 16.06%. The results demonstrated that the ratio of cells inside the pattern to the outside has increased from 0.42 to 5.22 after using the epoxy silane stamp method. These results are in line with the collagen immobilization results before and after the epoxy stamp method. Consequently, the epoxy silane stamp approach has effectively induced selective cell adhesion and proliferation within patterned areas by eliminating collagen from unfavorable regions. As we know from previous studies, matching cells morphology with the form of other cells is one of the main factors in their fate. After placing the stem cells into the mature cell patterns, the result shows that they can translate their morphology and go toward particular differentiation. The fundamental issue with the traditional imprinting technique is the lack of assurance about whether stem cells were exactly placed in the mature cell pattern. Hence, the distribution of the cells was completely arbitrary and caused unwanted cell fate. While applying the presented method in the current study has overcome this problem.

Besides, it observes that the number of attached cells on the selective collagen-immobilized samples in comparison with the random samples has decreased. Previously, many studies showed that the collagen coating on the PDMS substrate improves cell attachment and proliferation^30^. As a result, after removing collagen from a portion of the cell culture substrate, the number of connected cells fell, as did the number of viable cells, but according to the primary objective of the research, the cells were attached to the appropriate locations. Definitely, the issue of reducing the number of adherent cells is related to the specific template of the aligned cell-imprinted substrate. Whereas, random and high confluency cell-imprinted PDMS substrates that are normally used in cell-imprinted studies can address this problem.

For the first time, the collagen was immobilized selectively into the micro/nano cell-imprinted surface cavities. Furthermore, in previous studies, the epoxy-activated substrate was used to immobilize the proteins and enzymes^10–13^. Hence, introducing the concept of using an epoxy-activated substrate to remove the protein from undesired regions, obtained fascinating results. Kim et al. used the stamp method to remove RGD-phage from the ridge region of the PDMS mold containing microscale topographical patterns fabricated through photolithography. Their results showed an enhancement of cell adhesion and induction of cell alignment for the cardiomyocyte^31^. In none of the previous studies on the cell-imprinted substrate, the patterned region has not been selectively modified with protein for specific cell attachment. In previous studies, the optical contact lithography process modified the cell-imprinted surface. In their report, flat areas are covered with Polyethylene glycol (PEG) as a repelling molecule to prevent cell attachment to undesirable regions^32^. Despite achievements, this method needs two photolithography steps to construct the stimulated mask, which is a time-consuming and expensive procedure. In the present studies, the epoxy silane resin stamp process directly guides the cells for purposeful attachment into the patterned area. Therefore, the stamp method applied in the present study can recommend a simple and cost-effective method to use for all patterned substrates with different dimensions and topography. Moreover, by the immobilization of particular proteins instead of collagen, this method can offer unique advantages for diverse purposes.

## Conclusion

Biofunctionalization by proteins has proven to be an effective method to enhance cell attachment and proliferation. Many studies showed that collagen immobilization provides enhancement on cell adhesion and cell viability. Hence, we successfully presented a selective method for collagen immobilization on the desired region of cell-imprinted PDMS and demonstrated how cells respond to the selected collagen-immobilized substrate. To better display, the selective biofunctionalization of the cell-imprinted PDMS method, the aligned cell-imprinted PDMS was considered. The performance of the applied method on collagen immobilization and cell-PDMS integration were assessed by Coomassie brilliant blue staining and crystal violet staining. By applying the epoxy silane resin stamp method, the immobilized collagen was removed from the ridge of the PDMS substrate where there is no cell pattern cavity. Consequently, following cell seeding, it was discovered that the cells preferentially adhered to the substrate and matched the aligned cell-imprinted pattern. Due to entrapping stem cells in a cell-imprinted pattern or cultivating cells in a specific pattern, such as an aligned arrangement, this technique may be useful for future research on cell differentiation. Although selective protein immobilization into the concavity region of cell-imprinted PDMS could be achieved with microfluidic devices, with epoxy silane stamp method is very simple and cost-effective which allows us to reach selective cell attachment without using any additional cell adhesive molecules or microfluidic chips to pass cell flow through the micro-channels. Furthermore, microfluidic devices are useful for protein immobilization in special patterns, while the epoxy silane stamp method could be applied for all random and regular cell-imprinted substrates. Therefore, our approach could present some benefits for microfluidic studies and immobilization of other ECM proteins to specific targets like stem cell differentiation.

## Data Availability

All importance data generated or analyzed during this study are included in this published article and a limited number of others, if needed available from the corresponding author on reasonable request.
